# Gonadal Transcriptome Analysis and Sequence Characterization of Sex-Related Genes in *Cranoglanis bouderius*

**DOI:** 10.3390/ijms232415840

**Published:** 2022-12-13

**Authors:** Dongjie Wang, Zhengkun Pan, Guoxia Wang, Bin Ye, Qiujie Wang, Zhiheng Zuo, Jixing Zou, Shaolin Xie

**Affiliations:** 1 College of Marine Sciences, South China Agricultural University, Guangzhou 510642, China; 2 Key Laboratory of Animal Nutrition and Feed Science (South China) of Ministry of Agriculture, Guangzhou 510640, China; 3 Key Laboratory of Animal Breeding and Nutrition, Guangdong Public Laboratory of Animal Breeding and Nutrition, Institute of Animal Science, Guangdong Academy of Agricultural Sciences, Guangzhou 510640, China

**Keywords:** gonadal, *Cranoglanis bouderius*, transcriptome, molecular pathway, sex differentiation, sex-related genes

## Abstract

In China, the *Cranoglanis bouderius* is classified as a national class II-protected animal. The development of *C. bouderius* populations has been affected by a variety of factors over the past few decades, with severe declines occurring. Considering the likelihood of continued population declines of the *C. bouderius* in the future, it is critical to investigate the currently unknown characteristics of gonadal differentiation and sex-related genes for *C. bouderius* conservation. In this study, the Illumina sequencing platform was used to sequence the gonadal transcriptome of the *C. bouderius* to identify the pathways and genes related to gonadal development and analyze the expression differences in the gonads. A total of 12,002 DEGs were identified, with 7220 being significantly expressed in the ovary and 4782 being significantly expressed in the testis. According to the functional enrichment results, the cell cycle, RNA transport, apoptosis, Wnt signaling pathway, p53 signaling pathway, and prolactin signaling pathway play important roles in sex development in the *C. bouderius*. Furthermore, the sequence characterization and evolutionary analysis revealed that AMH, DAX1, NANOS1, and AR of the *C. bouderius* are highly conserved. Specifically, the qRT-PCR results from various tissues showed significant differences in AMH, DAX1, NANOS1, and AR expression levels in the gonads of both sexes of *C. bouderius*. These analyses indicated that AMH, DAX1, NANOS1, and AR may play important roles in the differentiation and development of *C. bouderius* gonads. To our best knowledge, this study is the first to analyze the *C. bouderius* gonadal transcriptome and identify the structures of sex-related genes, laying the foundation for future research.

## 1. Introduction

Generally, there are two kinds of biological sex determination: genetic sex determination (GSD) and environmental sex determination (ESD) [1,2,3]. GSD refers to sex determination by the organism’s chromosomes, whereas ESD refers to sex determination by environmental factors such as hormones [4], temperature [5], light intensity [6], pH [7], and so on. Fish, unlike other animals, have almost all the vertebrate sex chromosome types. There are eight types of fish chromosome systems that have been reported [8,9,10], indicating that GSD-dominated fish have a complex and diverse sex determination strategy [11]. The molecular mechanisms of sex regulation in fish are still unknown due to the complexity of the fish sex-determination system.

Despite the fact that basic information on sex determination pathways and genes is available for only a small percentage of fish, much progress has been made in cumulative research. A number of classical hermaphrodite-related pathways and genes have been identified in fish, including AMH in the transforming growth factor superfamily [12,13], DMRT1 in the Dmrt family [14], WN4 in the Wnt family [15], DAX1 in the nuclear receptor (NR) superfamily [16], the maternal effector gene nanos [17], and the nuclear receptor family’s AR [18]. Previous studies have shown that knocking down AMH activates the expression of foxl2 and cyp19a1a, leading to the reversal of male-to-female sexing in the *Odontesthes hatcheri* [19]. In *Oryzias latipes*, the DAX1 dose-dependently inhibits the transcriptional activation of cyp19a1a by sf1 and foxl2 [20]. Koprunner et al. demonstrated that the nanos gene is essential for the correct migration and viability of zebrafish PGCs by injecting nanos mRNA into embryos of cells lacking parental nanos [21]. In the absence of ligands, AR forms a complex with related molecular partners and enters the nucleus to recruit a number of factors to form a transcriptional enhancer complex, which in turn regulates the transcription of reproduction-related target genes [22]. These findings suggest that AMH, DAX1, NANOS1NANOS1, and AR play an important role in the regulation of sex determination.

*Cranoglanis bouderius* is classified under Siluriformes, Cranoglanididae, considered the sole representative of *Cranoglanis* [23,24]. It is an economic fish endemic to East Asia, found primarily in the Pearl River and Hainan Island basins of southern China, as well as the Red River basin of northern Vietnam, and has high nutritional and food value [25]. Over the past few decades, the *C. bouderius* wild population has declined significantly due to dam construction, overfishing, and environmental pollution, and it is now one of the vulnerable endangered species listed in the China Red Data Book of Endangered Animals [26]. Although some work has been done by other researchers and us on the habits, reproductive biology, and breeding techniques of this fish, and some results have been achieved, it has not yet made a breakthrough in large-scale breeding and breeding techniques to achieve an effective supplement to the wild population [27,28]. The lack of reproduction-related genetic information is an important factor limiting the conservation and sustainable use of *C. bouderius* resources.

In this study, we used the Illumina sequencing platform to construct and sequence the first cDNA library of *C. bouderius* testes and ovaries, and the data obtained may serve as a useful genetic resource for future reproductive control studies and for the selection of candidate genes related to sex determination and maintenance. In addition, we have cloned and obtained the full-length cDNA of AMH, DAX1, NANOS1, and AR of *C. bouderius* and characterized their structures for the first time. Finally, we describe in detail the expression characteristics of AMH, DAX1, NANOS1, and AR in different tissues of *C. bouderius*. These results are expected to provide guidance for research on the molecular breeding and sex-determination mechanisms of *C. bouderius*.

## 2. Results

### 2.1. Sequencing Quality Assessment

The gonads of the female and male groups were stained with hematoxylin-eosin (HE) and observed in tissue sections. Specifically, the spermatocysts are clearly visible in the testis and are filled with spermatocytes and spermatocytes. Similarly, oocytes of different developmental stages were seen in the ovaries, and their number and morphological structure were normal (Figure S1). A total of 46.51 Gb of clean data was obtained after the initial sequencing data quality control. The average base error rate of each library is less than 0.025%. The average Q20, Q30, and GC content of the samples is 98.19%, 94.80%, and 49.84%, respectively. The clean reads of each sample were mapped to the reference genome (unpublished), and the comparison results showed that the comparison rate of each sample was more than 93% (Table 1). These results suggested that the RNA sequencing produced high-confidence sequences.

### 2.2. Transcriptome Data Validation

**Figure 1:**
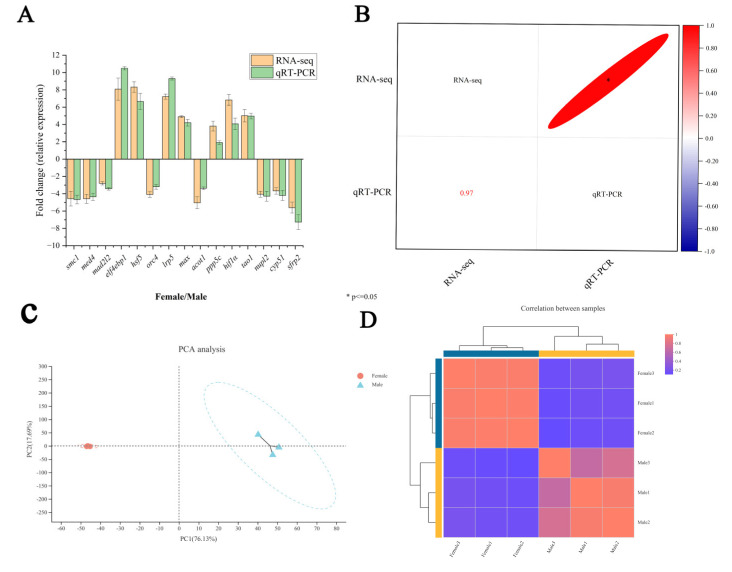
Transcriptome data validation. (**A**): Log_2_^(Fold change)^ analysis between female and male. (**B**): Pearson correlation analysis of qRT−PCR and RNA−Seq data. (**C**): PCA analysis of female and male data. (**D**): female and male correlation analysis.

To verify the accuracy of the RNA-Seq, a quantification of the expression levels of 15 randomly selected DEGs in the ovary and testis by qRT-PCR was conducted. As shown in Figure 1A, the expression patterns of the 15 DEGs matched well with the results in the RNA-Seq data. A Pearson correlation analysis showed that the qRT-PCR data of 15 DEGs were significantly and positively correlated with the transcriptome data (correlation coefficient = 0.97, *p* < 0.05) (Figure 1B). The above results indicate that the RNA-Seq data obtained in this study are reliable and can be used for subsequent analysis.

Overall, a total of 28,024 expressed genes were detected, including 21,914 known genes and 6110 unknown genes (Table S4). Based on the expression matrix, a PCA and correlation analysis between the samples were performed. The results of the PCA analysis showed that the samples of the male and female groups were well clustered, and individuals had little influence on the male and female groups (Figure 1C). The correlation analysis results showed that the correlation between the biological replicates between the samples was high (Figure 1D). These results show that the quality of each sample is consistent with the experimental design.

### 2.3. Analysis of Differentially Expressed Genes (DEGs)

**Figure 2:**
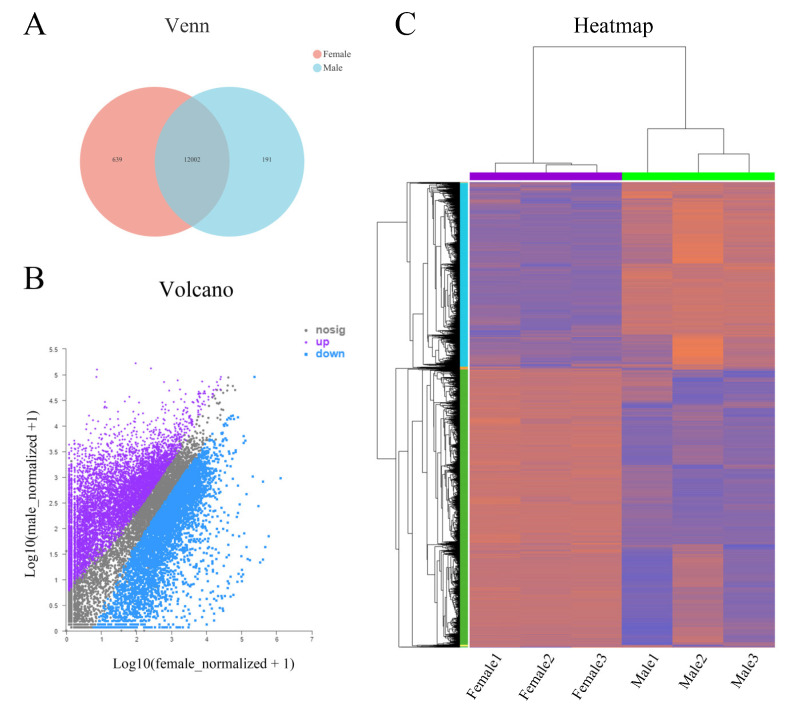
Differentially expressed genes (DEGs) between ovary and testis. (**A**): number of DEGs. (**B**): volcano maps of DEGs. (**C**): clustering of DEGs.

The differential expression analysis software DESeq2 was used to identify the DEGs of the co-expressed genes. A total of 12002 DEGs was differentially expressed in the testis as a control group (Figure 2A), with 7220 (60.16%) genes significantly highly expressed in the ovary and 4782 (39.84%) genes significantly highly expressed in the testis (Figure 2B). The clustering analysis showed that the clustering patterns of DEGs in the female and male samples were generally consistent, indicating that the expression patterns of DEGs in the female and male samples were similar (Figure 2C). These results suggest that the DEGs in the ovary or testis may have similar biological functions or be involved in similar biological processes.

### 2.4. Identification of Sex-Related DEGs

**Figure 3:**
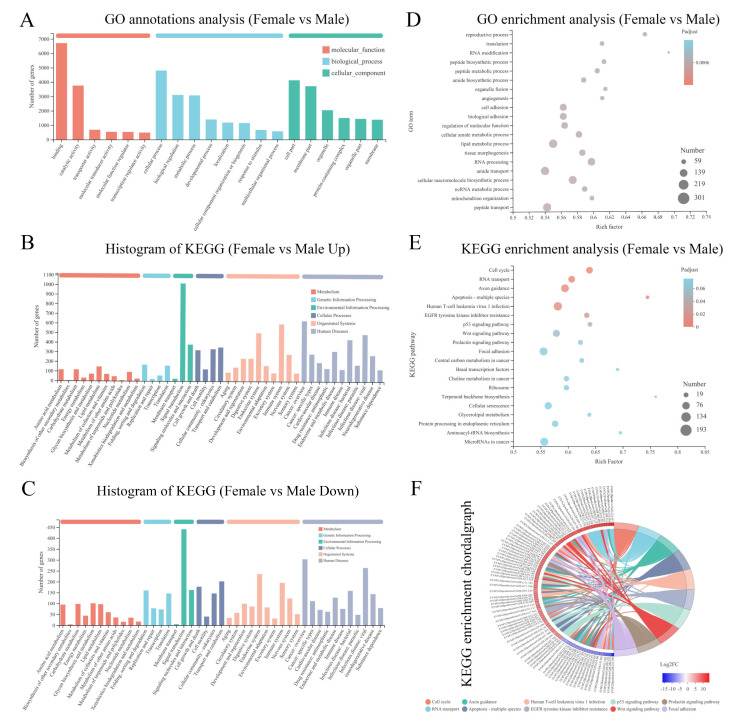
GO and KEGG analysis of DEGs. (**A**): GO annotation of responsive DEGs. (**B**): KEGG annotation of responsive DEGs (Up). (**C**): KEGG annotation of responsive DEGs (Down). (**D**): GO enrichment analysis. (**E**): KEGG enrichment analysis. (**F**): KEGG enrichment chordal graph.

The differentially expressed genes were annotated into the GO database, enriched to analyze their involvement in biological processes and functions in the gonads and screened for sex-linked genes. A total of 10,635 DEGs were annotated into 965 GO branches. The highest number of DEGs were annotated in three categories: cellular processes (4804 DEGs), biological regulation (3102 DEGs), and metabolic processes (3071 DEGs). A GO enrichment analysis revealed a total of five biological processes associated with sex, including the reproductive process (111 DEGs), enzyme-linked receptor protein-signaling pathway (132 DEGs), regulation of transcription by RNA polymerase II (257 DEGs), binding of sperm to zona pellucida (9 DEGs), and negative regulation of the reproductive process (4 DEGs) (Figure 3A,D).

The KEGG annotation results showed that a total of 8585 DEGs were annotated into 346 signaling pathways, of which up-regulated (ovarian high-expression) genes were annotated into 336 signaling pathways (Figure 3B), and down-regulated (testis high-expression) genes were enriched into 344 signaling pathways (Figure 3C). After screening for signaling pathways, a total of 25 gender-related signaling pathways were obtained (Table S5). In addition, DEGs were most enriched in the cell cycle, RNA transport, apoptosis, Wnt signaling pathway, p53 signaling pathway, and prolactin signaling pathway (Figure 3E,F). This suggests that these signaling pathways and enriched DEGs play important roles in sex determination, differentiation, and regulation of *C. bouderius*. In addition, several genes related to sex determination and gametogenesis in fish were screened (Table S6).

### 2.5. Sequence Characterization of Sex-Related Genes

**Figure 4:**
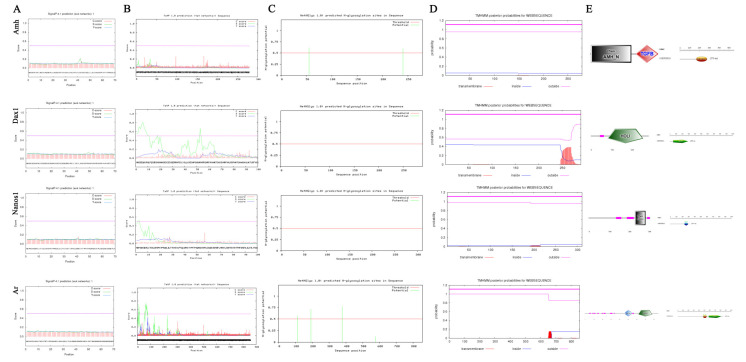
Sequence characterization of AMH, DAX1, NANOS1, and AR. (**A**): shear site; (**B**): signal peptide; (**C**): glycosylation sites; (**D**): transmembrane structural domains; (**E**): functional domain prediction.

As mentioned above, we found that the AMH, DAX1, NANOS1, and AR of *C. bouderius* have the property of being highly expressed in spermatozoa (Table S6). To further elucidate their structural and expression characteristics, we further explored their sequence features and tissue expression levels. The prediction results showed that AMH, DAX1, NANOS1, and AR proteins have no signal peptide and no shear site (Figure 4A,B). AMH protein has two (55 NSSG and 240 NRTL) potential N-glycosylation sites and AR protein has four (112 NHSS, 192 NRSS, 378 NPTC, and 576 NPSP) potential glycosylation sites, while DAX1 and NANOS1 proteins do not have glycosylation sites (Figure 4C). The results of the transmembrane structure prediction showed that the amino acids of AMH, DAX1, NANOS1, and AR proteins are located outside the membrane and have no transmembrane structural domains (Figure 4D). The functional domain prediction results indicated that the amino acid sites 169 to 279 of AMH are the TGF-β 2 functional structural domain, 53 to 290 of DAX1 are the NR LBD structural domain, 233 to 287 of NANOS1 are the zf-nanos structural domain, while 471 to 546 and 581 to 815 of AR are the ZnF-C4 and the HOLI structural domain, respectively (Figure 4E).

**Figure 5:**
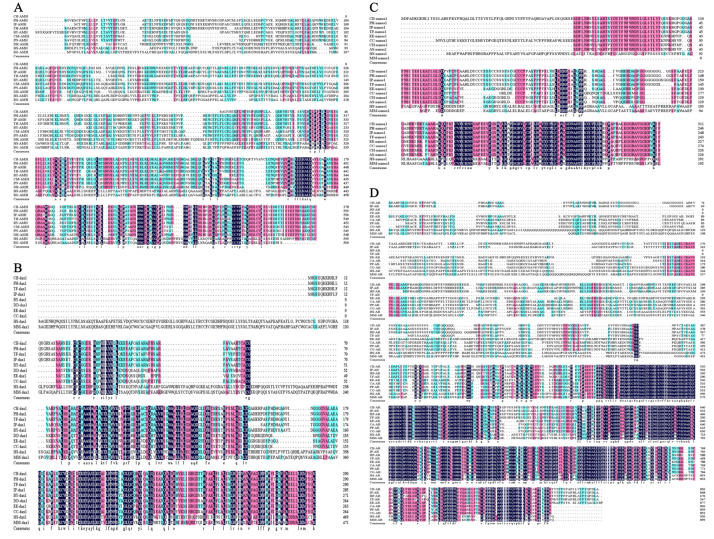
Comparison of the amino acid sequences of AMH, DAX1, NANOS1 and AR of *C. bouderius* with other species. CB: *C. bouderius*. PH: *Phypophthalmus*. IP: *Ictalurus punctatus*. TF: *Tachysurus fulvidraco*. BY: *Bagarius yarrelli*. CM: *Colossoma macropomum*. PN: *Pygocentrus nattereri*. DR: *Danio rerio*. HS: *Homo sapiens*. BG: *Bos grunniens*. Regions with the same color represent conserved sites of the sequence, and Black represents fully conserved loci. (**A**) Comparison of amino acid sequences of AMH in different species. (**B**) Comparison of amino acid sequences of DAX1 in different species. (**C**) Comparison of amino acid sequences of NANOS1 in different species. (**D**) Comparison of amino acid sequences of AR in different species.

An amino acid sequence alignment and analysis showed that the amino acid sequences of AMH, DAX1, NANOS1, and AR proteins are conserved in fish, with highly conserved regions mainly at the C-terminal (Figure 5). This may be an important factor in their ability to perform stable physiological functions in different fish species.

**Figure 6:**
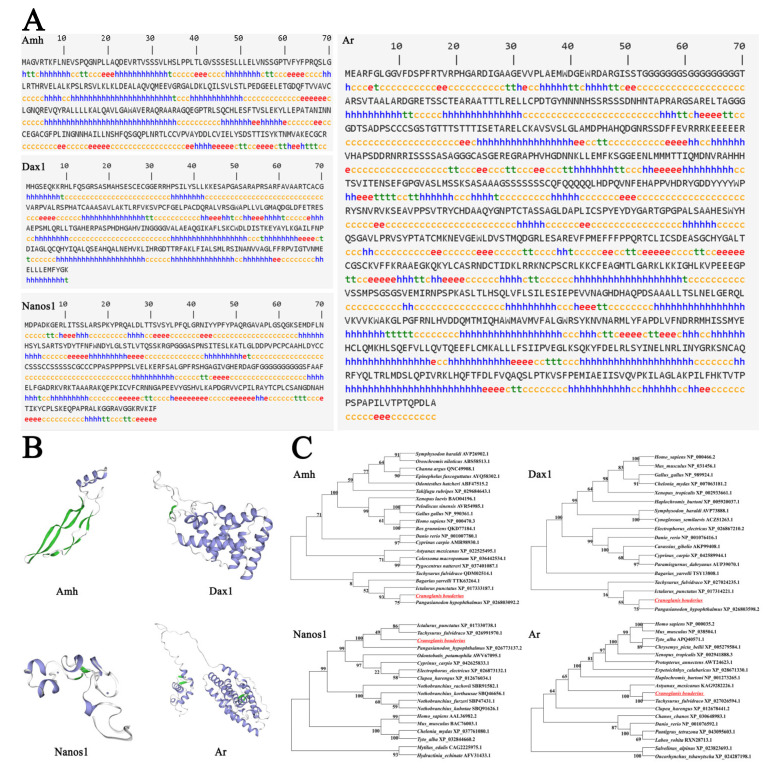
Secondary structure, tertiary structure, and phylogenetic tree of AMH, DAX1, NANOS1, and AR of *C. bouderius*. α-Helix(h), β-fold (e), β-Turning angle (t) and Irregular curl (c).

As shown in Figure 6, to elucidate the potential functional and evolutionary relationships of AMH, DAX1, NANOS1, and AR of *C. bouderius*, we predicted their secondary and tertiary structures and constructed molecular phylogenetic trees based on the amino acid sequences. The results show that the α-helix (h) accounted for 41.94%, β-fold (e) accounted for 14.70%, β-turn (t) accounted for 5.38%, and irregular curl (c) accounted for 37.99% of the AMH protein secondary structure (Figure 6A). The α-helix (h) accounted for 45.52%, the β-fold (e) accounted for 5.86%, the β-turn (t) accounted for 3.10%, and the irregular curl (c) accounted for 45.52% of the DAX1 protein secondary structure (Figure 6A). The α-helix (h) accounted for 22.19%, the β-fold (e) accounted for 17.36%, the β-turn (t) accounted for 4.82%, and the irregular curl (c) accounted for 55.63% of the DAX1 protein secondary structure (Figure 6A). The α-helix (h) accounted for 34.81%, the β-fold (e) accounted for 10.28%, the β-turn (t) accounted for 7.01%, and the irregular curl (c) accounted for 47.90% of the AR protein secondary structure (Figure 6A).

The results of the tertiary structure analysis showed that the tertiary structure of the AMH protein was modeled in the range of 187 to 289 with 33% coverage, 41% sequence similarity, and 43.01% sequence identity, which is the structure of AMH bound to AMHR2 ECD (Figure 6B). The tertiary structure of the DAX1 protein was modeled in the range of 78 to 290 with 76% coverage, 43% sequence similarity, and 49.77% sequence identity, which is the structure of DAX1 bound to LRH1 (Figure 6B). The tertiary structure of the NANOS1 protein was modeled in the range of 229 to 295 with 24% coverage, 42% sequence similarity, and 45.33% sequence identity, which is the structure of DAX1 bound to a zinc ion (Figure 6B). The tertiary structure of the AR protein was modeled in the range of 471 to 818 with 40% coverage, 33% sequence similarity, and 24.12% sequence identity, which is the structure of liver X nuclear-receptor beta (Figure 6B).

The molecular phylogenetic trees were constructed based on the amino acid sequences. The homology analysis showed that the AMH, DAX1, NANOS1, and AR of *C. bouderius* with *P. hypophthalmus*, *I. punctatus,* and *T. fulvidraco* were closest in kinship (Figure 6C). There is general agreement with the known taxonomic relationships between these species.

### 2.6. Sex-Related Gene Tissue Expression

**Figure 7:**
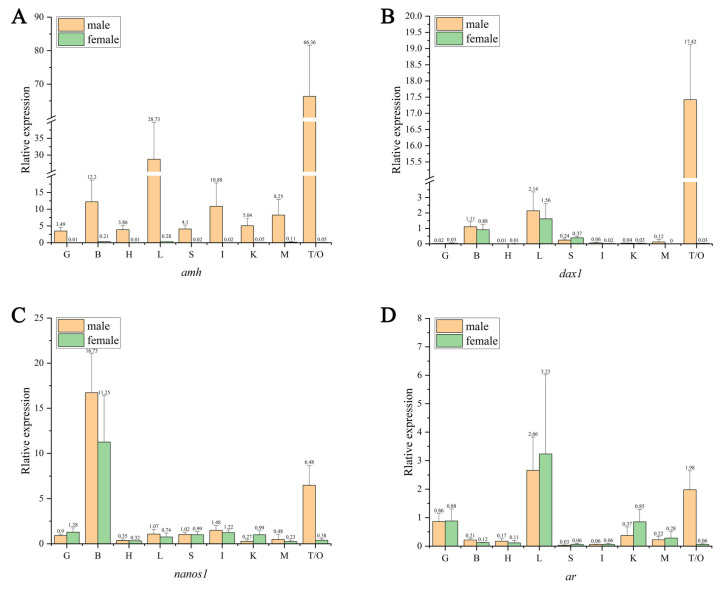
Expression of AMH, DAX1, NANOS1, and AR in various tissues of *C. bouderius*. G: gill; B: brain; H: heart; L: liver; S: spleen; I: intestine; K: kidney; M: muscle; T: testis; O: ovary.

The OD260/OD280 of total RNA was 1.8~2.0, and three bands of 28S, 18S, and 5S could be observed simultaneously under the gel imaging system, indicating that the extracted total RNA could be used for further analysis. The qRT-PCR results showed that the AMH was expressed only in the male *C. bouderius* and not in the female, and at the highest level in the male testis tissue, followed by lower levels in the liver, brain, intestine, and muscle, and the lowest levels in the kidney, spleen, heart, and gill (Figure 7A). The DAX1 was mainly expressed in the liver and testis, with the highest expression in the testis and lower or no expression in other tissues (Figure 7B). The NANOS1 was mainly expressed in the brain of both sexes and the testis of males, with the highest expression in the brain and testis, and low or no expression in other tissues (Figure 7C). The AR was most expressed in the liver of both females and males, with very low expression in the gonads of females and low or no expression in other tissues (Figure 7D). Notably, the screened sex-related genes were specifically expressed in the gonadal tissues of the testis and not in the ovary.

## 3. Discussion

In this study, the cDNA libraries of the testis and ovary of *C. bouderius* were constructed for the first time, with Q30 > 94.56% and Q20 > 98.05% of the sequences indicating the high quality of the data obtained in this study. The *C. bouderius* gonadal transcript sequences were found to be enriched in the GO database in the functional categories “cellular processes”, “cellular”, and “binding”, and, in the KEGG database, in “metabolism”, “genetic information processing”, “environmental information processing”, “cellular processes”, “organismal systems”, and “human diseases”. This is consistent with other fish gonadal RNA-Seq findings, indicating that the genes in these categories are functionally conserved [29,30,31,32].

In various vertebrates, dozens of effector pathways and genes involved in sex determination and gonadal development have been identified, including pathways involved in the Sox [33], Dmrt [34], Hox [35], and Wnt [36] gene families, as well as genes such as Sox9, Sox3, DAX1, AMH, Dmrt1, and Fgf9. The current research has demonstrated that gonadogenesis and development in fish are also co-regulated by these genes [37,38]. We performed a functional analysis of the DEGs, based on the known sequences in the database, and discovered that the DEGs were significantly enriched in the “reproductive process”, “ RNA modification”, “amide biosynthetic process”, “lipid metabolic process”, and “peptide transport processes”. The KEGG pathway analysis revealed that 25 of the 344 signaling pathways enriched were involved in gonadal differentiation and development in *C. bouderius*, including the TGF-β, Wnt, p53, mTOR, and GnRH signaling pathways, which have been implicated in gonadal differentiation and development in fish in previous studies [39,40,41]. This implies that the abundance of sex-related biological processes and metabolic pathway data obtained in this study will provide reliable basic data for future studies of sex-related genes in *C. bouderius*.

Studies have shown that AMH, DAX1, NANOS1, and AR play important roles in fish gonadal differentiation and development. The current study demonstrates that AMH has two structural domains, AMH-N and TGF-β [42]. TGF is a multifunctional peptide that not only regulates cell proliferation and differentiation but is also involved in GSD in fish [43]. Nuclear receptors (NRs) are a type of transcriptional regulator that regulate organismal homeostasis, reproduction, development, and metabolism. Typical NRs share a structure known as the HOLI structural domain. In this study, DAX1 contains a HOLI structural domain and an NR-LBD functional domain. Nanos is a highly conserved RNA-binding protein in higher eukaryotes that plays an important regulatory role in germ cell development and maintenance [44]. The NANOS1 predicted in this study has a zf-nanos (zinc-finger nanos-type) functional structural domain, which is consistent with other fish [45]. Similarly, AR has a structural domain called ZnF_C4 (zinc-finger c4-type). These findings suggest that AMH, DAX1, NANOS1, and AR are directly involved in the development, sexual differentiation, and maintenance of *C. bouderius’* gonadal organs [46]. The phylogenetic tree shows that AMH, DAX1, NANOS1 and AR cluster first with *Pangasianodon hypophthalmus*, *Ictalurus punctatus,* and *Pelteobagrus fulvidraco*, and then with other fishes, elucidating *C. bouderius*’ taxonomic status. These findings also support the hypothesis that AMH, DAX1, NANOS1, and AR of *C. bouderius* are evolutionarily conserved, possibly due to their important functions.

Despite the lack of Müllerian duct tissue in scleractinian fishes, the AMH is still important in fish gonads. For example, mutations in the AMH in zebrafish cause gonadal hypoplasia and dysfunction [47]. The AMH expression was highest in the testis of *C. bouderius*, indicating that the AMH still plays an important role in the spermathecae of *C. bouderius*. The DAX1 is responsible for X-linked congenital adrenal insufficiency and hypogonadism [48]. In addition, DAX1 expression gradually increases with gonad development in rainbow trout (*Oncorhynchus mykiss*), indicating that it plays an important role in testis development. In *C. bouderius*, DAX1 was also observed to be predominantly expressed in the testis, suggesting that DAX1 also plays an important role in the testis and development in *C. bouderius*. The complete sterility of NANOS1 mutants during zebrafish gonad development confirms that NANOS1 expression is required to maintain gonad development [49]. In this study, NANOS1 is mainly expressed in the brain and testis, and in combination with the results of structural prediction analysis of NANOS1, NANOS1 plays a major role in the development and maintenance of germ cells in *C. bouderius*. Male zebrafish with AR knockout have sterility, smaller testes, and impaired sperm development. Furthermore, peak AR expression occurred in zebrafish on days 16 and 22 after hatching. The AR is primarily expressed in the liver and testis of *C. bouderius*, which is consistent with Jorgensen et al. that AR plays an important role in gonadal differentiation and maintenance [50]. Finally, the broad conservation and differential tissue expression profiles of AMH, DAX1, NANOS1, and AR suggest that they are involved in gonadal differentiation and development in *C. bouderius* and that their specific functions need to be investigated further.

## 4. Materials and Methods

### 4.1. Experimental Fish and Sample Collection

*The C. bouderius* specimens (body weight: 851.13 ± 5.63 g, body length: 43.1 ± 1.6 cm) were collected in the Nanhai District (E: 113°9′23′′, N: 23°6′28′′), Foshan City, Guangdong Province, and immediately transported to the laboratory. All the experiments were performed in accordance with the Guidelines for the Care and Use of Laboratory Animals in China and approved by the Institutional Animal Care and Use Committee (IACUC), South China Agricultural University, Guangzhou, China. All the experimental fish were euthanized using 100 mg/L tricaine methanesulfonate (MS-222, Sigma, Milwaukee, USA), and an incision for gender determination was made in the abdomen. Gonad samples were collected and divided into three parts. The samples of the histology observations were fixed in 4% paraformaldehyde. The remaining samples were snap-frozen in liquid N_2_ and stored at –80 °C for later transcriptome analysis and RNA quantification. Meanwhile, the gills, brains, hearts, livers, spleens, intestines, kidneys, and muscles of the females and males were collected for subsequent tissue expression analysis of the sex-related genes.

### 4.2. RNA Extraction, Library Building and Sequencing

The developmental stages of the gonads of *C. bouderius* were confirmed by histological examination by an automatic digital slide scanning system (M8, Precipoint, Germany). Three testis tissues (Male1, Male2, and Male3) and three ovarian tissues (Female1, Female2, and Female3) with identical developmental stages were randomly selected and used for transcriptome sequencing (Figure S1). Total RNA was extracted with TRIzol reagent (Invitrogen) according to the manufacturer’s protocol and then treated with RNase-free DNase I (Takara, Tokyo, Japan) to prevent contamination. The concentration and quality of total RNA were determined by the NanoDrop 2000 (Thermo, Pittsburg, PA, USA) and the Agilent 2100 Bioanalyzer (Agilent, San Diego, CA, USA). The total amount of RNA in a single library was ≥ 1ug; the concentration was ≥ 35 ng/microliter, OD260/280 ≥ 1.8, and OD260/230 ≥ 1.0. The mRNA libraries of the Male1, Male2, Male3, Female1, Female2, and Female3 were created using a TruSeq RNA Sample Prep Kit (Illumina, USA) according to the manufacturer’s protocol and were sequenced by an Illumina NovaSeq 6000 system.

### 4.3. Transcriptome Data Validation

In order to verify the accuracy of the transcriptome data, 15 DEGs were randomly selected, and their expression levels in the gonads of *C. bouderius* were detected by quantitative real-time PCR (qRT-PCR). The primers for DEGs are listed in Table S1. The qRT-PCR of the sex-related genes was carried out on a CFX Connect Real-Time System (Bio-Rad) using THUNDERBIRD SYBR. reaction system: 2× SYBR Green Pro Taq HS Premix, 5 μL; cDNA (<100 ng), 1μL; Primer F (10 μM), 0.2 μL; Primer R (10 μM), 0.2 μL; RNase-free water, up to 10μL. qRT-PCR parameters: 95 ℃ for 30s; followed by 40 cycles at 95 °C for 5 s, 60 °C for 30 s; 72 °C for 30 s, and a final cycle of 72 °C for 7 min. Relative gene expression levels were normalized against the expression level of β-actin. The CFX Manager software was used to automatically analyze the CT value and baseline of the results of the qRT-PCR.

### 4.4. Identification and Functional Annotation of Differentially Expressed Genes (DEGs)

In order to ensure the accuracy of the subsequent bioinformatics analysis, SeqPrep and sickle software were used to filter the original sequencing data to obtain high-quality sequencing data (clean data). Then, the Q20, Q30, and GC contents of the clean data were calculated. The clean data were mapped to the genome of *C. bouderius* (PRJNA874211) using HISAT2-software. Based on the reference genome, the mapped reads were assembled and spliced using StringTie software. To annotate these UniGene functions, the mapped reads were annotated in the NR, Swiss-Prot, Pfam, eggNOG, GO, and KEGG databases. The quantitative software, RSEM, was used to quantify the expression levels of the genes and transcripts. The Per Kilobase Million and TPM algorithms were used to normalize the mRNA expression levels. Based on the quantitative expression results, DESeq2 was used to analyze the differential genes between groups. The screening threshold for differentially expressed genes was *p*-adjusted <0.05 and | log_2_ ^(fold change)^ | ≥ 1 and corrected for multiple testing using the BH method. The functional annotations of the DEGs were performed via the Kyoto Encyclopedia of Genes and Genomes (KEGG) pathway enrichment analysis.

### 4.5. Sequence Characterization of Sex-Related Gene

According to the sex-related genes screened by the transcriptome data, four highly expressed genes in the testis, AMH, DAX1, NANOS1, and AR, were selected for sequence characterization analysis. By aligning with the reference genome, we obtained AMH, DAX1, NANOS1 and AR gene sequences, CDS regions, and coding amino acid sequence information (Table S2). For these sequences, a SignalP 4.1 Server and TatP 1.0 Server software were used to predict protein signal peptides and cleavage sites, respectively. NetNGlyc 1.0 Server software was used to predict glycosylation sites. The TMHMM Server 2.0 software was used to predict protein transmembrane regions. The SMART and PROSIT tools were used to predict the conserved domains of amino acid sequences and their functional domain sites, respectively. Finally, SOPMA and SWISS-MODEL were used to predict the secondary and tertiary structures of the proteins, respectively. The sequences of species for the phylogenetic analysis were obtained from NCBI (Available online: https://www.ncbi.nlm.nih.gov). Phylogenetic trees based on amino acid sequences were constructed using the MEGA (V 7.0.25, Mega Limited, Auckland, New Zealand) software (neighbor-joining method).

### 4.6. Sex-Related Gene Tissue Expression Analysis

To clarify the expression of the sex-related genes AMH, DAX1, NANOS1 and AR in different tissues of *C. bouderius*, we extracted the total RNAs of the gill (G), brain (B), heart (H), liver (L), spleen (S), intestine (I), kidney (K), muscle (M), and gonads (testis/ovary, T/O). Primer sequences for the sex-related genes used for the qRT-PCR are shown in Table S3. β-Actin was used as an internal control to normalize the experimental data of the mRNA qRT-PCR.

### 4.7. Statistical Analysis

All the experiments were carried out at least in triplicate and the values are expressed as the mean ± S.E.M. The statistical analyses for this study were performed using SPSS statistical package version 26.0 (SPSS, Inc., Chicago, IL, USA) and OriginPro 2022 (Available online: www.OriginLab.com). Differences were determined by an analysis of variance (ANOVA). The qRT-PCR data were analyzed using the 2^−ΔΔCt^ method, and *p* < 0.05 was considered statistically significant.

## 5. Conclusions

In this study, the cDNA libraries of the ovaries and testis of *C. bouderius* were constructed for the first time, and the transcriptomes were sequenced; 12,002 DEGs were obtained after splicing and assembly, which enriched the genetic resources of *C. bouderius*. A number of pathways (25) and genes (513) involved in the development and differentiation of *C. bouderius* gonads were identified by comparing ovarian and testis DEGs. Based on the screening results, we analyzed the amino acid sequence characteristics of the AMH, DAX1, NANOS1, and AR using bioinformatic analysis software and quantified the tissue expression distribution of *C. bouderius* using a qRT-PCR. This study lays the foundation for further functional studies of sex-related genes and the development of sex-related molecular markers in *C. bouderius*.

## Figures and Tables

**Table 1:** Transcriptome sequencing data statistics.

Sample	Male 1	Male 2	Male 3	Female 1	Female 2	Female 3
Raw reads	50,755,254	44,882,700	54,264,414	51,980,490	59,555,972	56,712,898
Raw bases (GB)	7.66	6.77	8.19	7.84	8.99	8.56
Clean reads	50,230,124	44,382,438	53,706,854	51,603,750	59,081,800	56,248,822
Clean bases (GB)	7.38	6.50	7.89	7.64	8.76	8.34
Error rate (%)	0.0246	0.0246	0.0247	0.0241	0.0241	0.0244
Q20 (%)	98.11	98.07	98.05	98.34	98.34	98.21
Q30 (%)	94.64	94.63	94.56	95.11	95.11	94.77
GC (%)	49.04	49.1	49.98	50.19	50.26	50.45
Mapped	46,878,397	41,483,671	50,154,719	49,439,775	56,398,939	53,722,386
Mapped (%)	93.33	93.47	93.39	95.81	95.46	95.51
Uniquely mapped	43,477,040	38,131,053	45,239,980	44,625,697	51,147,482	48,902,184
Uniquely mapped (%)	86.56	85.91	84.24	86.48	86.57	86.94
Clean reads	50,230,124	44,382,438	53,706,854	51,603,750	59,081,800	56,248,822

## Data Availability

The authors declare that the data supporting the findings of this study are available within the article and its Supplementary Information Files.

## Supplementary Materials

The following supporting information can be downloaded at: https://www.mdpi.com/article/10.3390/ijms232415840/s1, Figure S1: Histological structure of the testis and ovary of *Cranoglanis bouderius*; Table S1: Sequences of primers; Table S2: Sequence information of *amh*, *dax1*, *nanos1* and *ar*; Table S3: Sequences of primers; Table S4: Expressed genes; Table S5: Gender-related signaling pathways; Table S6: Annotation information of sex-related genes.

## References

[1] Strussmann C.A., Yamamoto Y., Hattori R.S., Fernandino J.I., Somoza G.M. (2021). Where the Ends Meet: An Overview of Sex Determination in Atheriniform Fishes. Sex. Dev..

[2] Bokony V., Milne G., Pipoly I., Szekely T., Liker A. (2019). Sex ratios and bimaturism differ between temperature-dependent and genetic sex-determination systems in reptiles. BMC Evol. Biol..

[3] Muralidhar P., Veller C. (2018). Sexual antagonism and the instability of environmental sex determination. Nat. Ecol. Evol..

[4] Nakamura M. (2013). Is a Sex-Determining Gene(s) Necessary for Sex-Determination in Amphibians? Steroid Hormones May Be the Key Factor. Sex. Dev..

[5] Wiggins J.M., Santoyo-Brito E., Scales J.B., Fox S.F. (2020). Gene Dose Indicates Presence of Sex Chromosomes in Collared Lizards (*Crotaphytus collaris*), a Species with Temperature-Influenced Sex Determination. Herpetologica.

[6] Corona-Herrera G.A., Arranz S.E., Martinez-Palacios C.A., Navarrete-Ramirez P., Toledo-Cuevas E.M., Valdez-Alarcon J.J., Martinez-Chavez C.C. (2018). Experimental evidence of masculinization by continuous illumination in a temperature sex determination teleost (Atherinopsidae) model: Is oxidative stress involved?. J. Fish Biol..

[7] Kanaiwa M., Harada Y. (2008). Collapse of one-locus two-allele sex determining system by releasing sex-reversed hatchery fish. Rev. Fish. Sci..

[8] Andreata A.A., do Almeida-Toledo L., Oliveira C., de Toledo Filho S. (1993). Chromosome studies in hypoptopomatinae (Pisces, Siluriformes, Loricariidae). II. ZZ/ZW sex-chromosome system, B chromosomes, and constitutive heterochromatin differentiation in Microlepidogaster leucofrenatus. Cytogenet. Cell Genet..

[9] Nakamura D., Wachtel S.S., Kallman K. (1984). H-Y antigen and the evolution of heterogamety. J. Hered..

[10] Tave D. (1986). Genetics for Fish Hatchery Managers.

[11] Han C., Zhu Q.Y., Zhou X.N., Ouyang H.F., Han L.Q., Chen J.H., Li S.S., Li G.F., Lin H.R., Zhang Y. (2020). A PCR-based genetic sex identification method in spotted mandarin fish (*Siniperca scherzeri*) and big eye mandarin fish (*Siniperca kneri*). Aquac. Rep..

[12] Liu X.Y., Dai S.F., Wu J.H., Wei X.Y., Zhou X., Chen M.M., Tan D.J., Pu D.Y., Li M.H., Wang D.S. (2022). Roles of anti-Mullerian hormone and its duplicates in sex determination and germ cell proliferation of Nile tilapia. Genetics.

[13] Han Y.L., Zhao M., Wang L., Yu Z.S., Wang J., Yu Q., Xiao L., Lu M.W., Li S.S., Zhang Y. (2019). Overexpression of Anti-mullerian Hormone Gene *in vivo* Affects Gonad Sex Differentiation in Undifferentiated Orange-Spotted Groupers (*Epinephelus coioides*). Front. Endocrinol..

[14] Dong J., Li J., Hu J., Sun C., Tian Y., Li W., Yan N., Sun C., Sheng X., Yang S. (2020). Comparative Genomics Studies on the dmrt Gene Family in Fish. Front. Genet..

[15] Farhadi A., Fang S., Zhang Y., Cui W., Fang H., Ikhwanuddin M., Ma H. (2021). The significant sex-biased expression pattern of Sp-Wnt4 provides novel insights into the ovarian development of mud crab (Scylla Paramamosain). Int. J. Biol. Macromol..

[16] Xia X., Huo W., Wan R., Wang P., Chang Z. (2018). Cloning, characterization and function analysis of DAX1 in Chinese loach (*Paramisgurnus dabryanus*). Genetica.

[17] Tan X.G., Sui Y.L., Li M.J., Jiao S., Wu Z.H., You F. (2020). Characterization of nanos1 Homolog in the Olive Flounder, *Paralichthys olivaceus* (Temminck & Schlegel, 1846). Turk. J. Fish Quat. Sci..

[18] Golshan M., Alavi S.M.H. (2019). Androgen signaling in male fishes: Examples of anti-androgenic chemicals that cause reproductive disorders. Theriogenology.

[19] Hattori R.S., Murai Y., Oura M., Masuda S., Majhi S.K., Sakamoto T., Fernandino J.I., Somoza G.M., Yokota M., Strussmann C.A. (2012). A Y-linked anti-Mullerian hormone duplication takes over a critical role in sex determination. Proc. Natl. Acad. Sci. USA.

[20] Kurokawa H., Saito D., Nakamura S., Katoh-Fukui Y., Ohta K., Baba T., Morohashi K.I., Tanaka M. (2007). Germ cells are essential for sexual dimorphism in the medaka gonad. Proc. Natl. Acad. Sci. USA.

[21] Koprunner M., Thisse C., Thisse B., Raz E. (2001). A zebrafish nanos-related gene is essential for the development of primordial germ cells. Genes Dev..

[22] Black B.E., Paschal B.M. (2004). Intranuclear organization and function of the androgen receptor. Trends Endocrinol. Metab..

[23] Yang L., Liu C.X., He S.P. (2009). Threatened fishes of the world: *Cranoglanis bouderius* (Richardson, 1846) (Cranoglanididae). Environ. Biol. Fishes.

[24] Diogo R., Chardon M., Vandewalle P. (2002). Osteology and myology of the cephalic region and pectoral girdle of the Chinese catfish *Cranoglanis bouderius*, with a discussion on the autapomorphies and phylogenetic relationships of the Cranoglanididae (Teleostei:Siluriformes). J. Morphol..

[25] Peng Z.G., Wang J., He S.P. (2006). The complete mitochondrial genome of the helmet catfish *Cranoglanis bouderius* (Silurifonnes: Cranoglanididae) and the phylogeny of otophysan fishes. Gene.

[26] Yue P., Chen Y., Wang S. (1998). Pisces. China Red Data Book of Endangered Animals.

[27] Xie S.L., Zhou A.G., Feng Y.Y., Wang Z.L., Fan L.F., Zhang Y., Zeng F., Zou J.X. (2019). Effects of fasting and re-feeding on mstn and mstnb genes expressions in *Cranoglanis bouderius*. Gene.

[28] Chen J.T., Zhou A.G., Xie S.L., Wang C., Lv Z.J., Zou J.X. (2016). Comparative Proteomic Identification of Mature and Immature Sperm in the Catfish *Cranoglanis bouderius*. PLoS ONE.

[29] Tang Y., Chen J.Y., Ding G.H., Lin Z.H. (2021). Analyzing the gonadal transcriptome of the frog Hoplobatrachus rugulosus to identify genes involved in sex development. BMC Genom..

[30] Zhang Y., Waiho K., Ikhwanuddin M., Ma H.Y. (2021). Identification of Sex-Related Genes from the Three-Spot Swimming Crab *Portunus sanguinolentus* and Comparative Analysis with the Crucifix Crab *Charybdis feriatus*. Animals.

[31] Wu Y.P., Zhao X.Y., Chen L., Wang J.H., Duan Y.Q., Li H.Y., Lu L.Z. (2020). Transcriptomic Analyses of the Hypothalamic-Pituitary-Gonadal Axis Identify Candidate Genes Related to Egg Production in Xinjiang Yili Geese. Animals.

[32] Cui W.X., Yang Q., Zhang Y., Farhadi A., Fang H., Zheng H.P., Li S.K., Zhang Y.L., Ikhwanuddin M., Ma H.Y. (2021). Integrative Transcriptome Sequencing Reveals the Molecular Difference of Maturation Process of Ovary and Testis in Mud Crab *Scylla paramamosain*. Front. Mar. Sci..

[33] Hu Y.C., Wang B.Z., Du H.J. (2021). A review on sox genes in fish. Rev. Aquac..

[34] Xu S., Zhang S., Zhang W., Liu H., Wang M., Zhong L., Bian W., Chen X. (2022). Genome-Wide Identification, Phylogeny, and Expression Profile of the Dmrt (Doublesex and Mab-3 Related Transcription Factor) Gene Family in Channel Catfish (*Ictalurus punctatus*). Front. Genet..

[35] Pascual-Anaya J., Sato I., Sugahara F., Higuchi S., Paps J., Ren Y.D., Takagi W., Ruiz-Villalba A., Ota K.G., Wang W. (2018). Hagfish and lamprey Hox genes reveal conservation of temporal colinearity in vertebrates. Nat. Ecol. Evol..

[36] Safian D., Bogerd J., Schulz R.W. (2019). Regulation of spermatogonial development by Fsh: The complementary roles of locally produced Igf and Wnt signaling molecules in adult zebrafish testis. Gen. Comp. Endocrinol..

[37] Chao Q., Shen F., Xue Y., Wu J., Zhang J. (2020). Cbx2, a PcG Family Gene, Plays a Regulatory Role in Medaka Gonadal Development. Int. J. Mol. Sci..

[38] Castaneda-Cortes D.C., Fernandino J.I. (2021). Stress and sex determination in fish: From brain to gonads. Int. J. Dev. Biol..

[39] Chae M., Bae I.H., Lim S., Jung K., Roh J., Kim W. (2021). AP Collagen Peptides Prevent Cortisol-Induced Decrease of Collagen Type I in Human Dermal Fibroblasts. Int. J. Mol. Sci..

[40] He Z., Deng F.Q., Yang D.Y., He Z.D., Hu J.X., Ma Z.J., Zhang Q., He J.Y., Ye L.J., Chen H.J. (2022). Crosstalk between sex-related genes and apoptosis signaling reveals molecular insights into sex change in a protogynous hermaphroditic teleost fish, ricefield eel *Monopterus albus*. Aquaculture.

[41] Zohar Y., Zmora N., Trudeau V.L., Munoz-Cueto J.A., Golan M. (2022). A half century of fish gonadotropin-releasing hormones: Breaking paradigms. J. Neuroendocrinol..

[42] Munsterberg A., Lovell-Badge R. (1991). Expression of the mouse anti-mullerian hormone gene suggests a role in both male and female sexual differentiation. Development.

[43] Hinck A.P., Archer S.J., Qian S.W., Roberts A.B., Sporn M.B., Weatherbee J.A., Tsang M.L., Lucas R., Zhang B.L., Wenker J. (1996). Transforming growth factor beta 1: Three-dimensional structure in solution and comparison with the X-ray structure of transforming growth factor beta 2. Biochemistry.

[44] Bourguet W., Germain P., Gronemeyer H. (2000). Nuclear receptor ligand-binding domains: Three-dimensional structures, molecular interactions and pharmacological implications. Trends Pharmacol. Sci..

[45] Tao W.J., Yuan J., Zhou L.Y., Sun L.N., Sun Y.L., Yang S.J., Li M.H., Zeng S., Huang B.F., Wang D.H. (2013). Characterization of Gonadal Transcriptomes from Nile Tilapia (*Oreochromis niloticus*) Reveals Differentially Expressed Genes. PLoS ONE.

[46] Claessens F., Gewirth D.T., McEwan I.J. (2004). DNA recognition by nuclear receptors. Essays in Biochemistry: Nuclear Receptor Superfamily.

[47] Zhang Z.W., Zhu B., Chen W.T., Ge W. (2020). Anti-Mullerian hormone (Amh/*amh*) plays dual roles in maintaining gonadal homeostasis and gametogenesis in zebrafish. Mol. Cell. Endocrinol..

[48] Burris T.P., Guo W., Le T., McCabe E.R. (1995). Identification of a putative steroidogenic factor-1 response element in the DAX-1 promoter. Biochem. Biophys. Res. Commun..

[49] Draper B.W., McCallum C.M., Moens C.B. (2007). nanos1 is required to maintain oocyte production in adult zebrafish. Dev. Biol..

[50] Jorgensen A., Andersen O., Bjerregaard P., Rasmussen L.J. (2007). Identification and characterisation of an androgen receptor from zebrafish Danio rerio. Comp. Biochem. Physiol. C Toxicol. Pharmacol..

