# Supplementary material for: Gonadal Transcriptome Analysis and Sequence Characterization of Sex-Related Genes in Cranoglanis bouderius
Source: Int J Mol Sci. 2022 Dec 13;23(24):15840. doi: 10.3390/ijms232415840 (PMC9779447; doi:10.3390/ijms232415840)
Supplement: Supplementary file 1 [file ijms-23-15840-s001.zip › table S1 Sequences of primers..pdf]

Supplemental Table S1. Sequences of primers.

Primers	Sequences (5’ →3’)
β-actin-F	ATGAAGATCCTGACCGAGAGAGGCTAC
β-actin-R	GCCAATGGTGATGACCTGTCCG
evm.TU. Contig12.4-F	AGCGAGTACAGCATCCTTTC
evm.TU. Contig12.4-R	GCTTCTGGAGTGTCTCCCAT
evm.TU. Contig154.2-F	ACTGACCTTGAGATGCGTAG
evm.TU. Contig154.2-R	CTCATTCTCCTTATTGTGCC
evm.TU. LG21.491-F	TCCAAAAGAGGAAGAAATAC
evm.TU. LG21.491-R	GTTGTCTAAAACAGCATCAC
evm.TU. Contig213.2-F	CTTGGGCAGTAGAAAGAGCA
evm.TU. Contig213.2-R	GCATGGTTGTTACCGTTGAT
evm.TU. LG30.278-F	ACAACCCGAACTTCAAACG
evm.TU. LG30.278-R	CGGATAATGGAAATGCCTC
evm.TU. LG22.331-F	CCTGGAATCACAGTACAAGC
evm.TU. LG22.331-R	CAACGTAGCAGCATAGTTTT
evm.TU. LG18.427-F	GAGACACCACGAGGTTTGCC
evm.TU. LG18.427-R	GAAGAGCCCTGCCACGACA
evm.TU. Contig293.2-F	GGCTGGATGTGAGCACGAT
evm.TU. Contig293.2-R	CGCTGCGGAGGGAAGAAA
evm.TU. Contig293.6-F	TGGGCTTGTTCTGGTCTATGCTG
evm.TU. Contig293.6-R	CGTTGGTGGCTTCAGTGAGGAT
evm.TU. Contig303.3-F	TGACCAGATGGGTAACAAGGGAG
evm.TU. Contig303.3-R	GGCTTAACATTCGGATGAGGCAC
evm.TU. LG08.21-F	GCAGACCGCACCTGAGAAGACT
evm.TU. LG08.21-R	GAGGGAAGCATGACATCATTGTAGAG
evm.TU. Contig331.1-F	CCCCAACTCCATCACCGAATC
evm.TU. Contig331.1-R	CGCACGACGAGGAAGAGGA
evm.TU. Contig332.5-F	TTACGGAGATAAATGCTGGAATGAAC

evm.TU. Contig332.5-R	GGAGAACGATGAGGGCTGGAC
evm.TU. Contig332.6-F	TCATTGAAGAAGAAACGAGGCAGTA
evm.TU. Contig332.6-R	CAGGCAGCGTGGGTAAATCC
evm.TU. Contig345.4-F	CGCTCTTGCTCGGCATTTCT
evm.TU. Contig345.4-R	TGGCACTCGGTACGGGTCTG

F: (sense primer); R: (antisense primer).
