# Supplementary material for: Gonadal Transcriptome Analysis and Sequence Characterization of Sex-Related Genes in Cranoglanis bouderius
Source: Int J Mol Sci. 2022 Dec 13;23(24):15840. doi: 10.3390/ijms232415840 (PMC9779447; doi:10.3390/ijms232415840)
Supplement: Supplementary file 1 [file ijms-23-15840-s001.zip › table S2 sequence information of AMH, DAX1, NANOS1 and AR.pdf]

amh

Gene ID：evm.TU.LG21.491strand：+start：17832766end：17836251length：3486

Transcript ID：evm.model.LG21.491 start：17833436 end：17835413 length：840

CDS (evm.model. LG21.491) Length:840 type:complete

ATGGCTGGAGTAAGGACGAAGTTCTTAAATGAAGTCTCCCCTCAGGGAAAT
CCACTACTTGCACAAGATGAGGTCAGGACAGTCAGCTCGAGTGTTCTTCAC
TCTCTGCCCCCTTTAACTCTTGGAGTGTCTTCAAGCGAGTCTCTTCTTCTTG
AGCTGGTCAATTCCTCAGGCCCAACTGTGTTCTACTTCCCCCGCCAAAGCT
TGGGACTGAGGACCCACCGAGTGGAGCTGGCCCTAAAGCCATCACTGCGC
TCTGTGTTGAAATTAAAACTAGATGAGGCTTTAGCCCAAGTGCAGATGGAG
GAAGTTGGACGTGGTGCGCTAGATAAGCTGCAGATTCTCAGTGTTCTCAGT
ACACTTCCTGAAGATGGAGAGGAGCTGGAAACAGGTGATCAGTTTACTGT
TGTTGCTGTCTGTTTGGGGAACCAACGTGAGGTCCAATACAGAGCCCTCTT
ACTGCTGAAGGCCTTACAGGCTGTGCTAGGTGCTTGGGCAGTAGAAAGAG
CACAGAGGGCAGCCCGAGCTGGTCAGGAAGGACCCACAAGGCTTAGTCA
GTGTCATCTTGAGAGTTTCACTGTGTCTCTGGAGAAGTACCTGTTGGAACC
TGCCACGGCCAACATTAACAACTGTGAAGGAGCATGTGGATTCCCCCTGAT
CAACGGTAACAACCATGCCATCCTATTGAACAGTCACTTCCAGAGTGGACA
ACCTCTAAATCGTACACTCTGCTGTGTGCCCGTGGCATATGATGACCTATGT
GTGATTGAGCTGTACAGTGATTCCACCACCATTTCTTACAAGACCAACATG
GTAGCCAAGGAGTGTGGATGTCGCTGA

protein(evm.model.LG21.491) Length：279

MAGVRTKFLNEVSPQGNPLLAQDEVRTVSSSVLHSLPPLTLGVSSSESLLLELV
NSSGPTVFYFPRQSLGLRTHRVELALKPSLRSVLKLKLDEALAQVQMEEVGR
GALDKLQILSVLSTLPEDGEELETGDQFTVVAVCLGNQREVQYRALLLLKALQ
AVLGAWAVERAQRAARAGQEGPTRLSQCHLESFTVSLEKYLLEPATANINNCE
GACGFPLINGNNHAILLNSHFQSGQPLNRTLCCVPVAYDDLCVIELYSDSTTIS
YKTNMVAKECGCR

dax1

Gene ID：evm.TU.LG22.331strand：-start：16556084end：16557261length：1178

Transcript ID：evm.model.LG22.331 start：16556084 end：16557261 length：873

CDS (evm.model. LG22.331) Length:873 type:complete

ATGCATGGAAGTGAGCAGAAGAAGAGACATCTGTTCCAGTCAGGACGCTC
GGCGAGCATGGCTCATTCGGAGAGTTGTGAGTGCGGCGGCGAGCGGCGCC
ACCCGAGCATTCTCTACAGCCTGCTGAAGAAGGAGAGCGCGCCGGGCGCG
TCCGCGCGAGCGCCTCGCAGCGCCAGGTTCGCCGTGGCGGCGCGCACGTG
CGCGTGCGGTGTCGCGCGGCCGGTAGCGCTGCGCTCTCCGCACGCAACGT
GCGCCGCGGCCTCGGCTGTGCTCGCCAAGACGCTGCGCTTCGTCAAGAGC
GTGCCGTGTTTCGGTGAGCTGCCCGCCTGCGACCAGCGCGCTCTGGTGCG
AAGTGGCTGGGCGCCGTTGCTTGTGCTCGGAATGGCGCAGGACGGACTGG
ACTTCGAGACGCGCGAGAGCGCCGAGCCCAGCATGCTGCAGCGCCTCCTC
ACCGGCGCGCACGAGCGGCCCGCGAGCCCGCACGACCACGGCGCTCACG
TCATTAACGGCGGCGGAGGGGTGGCGCTGGCCGAGGCGCAGGGCATTAAA
GCGTTCCTCAGCAAATGCTGGGACCTCGACATCAGCACCAAGGAATACGCC
TACTTAAAGGGAGCCATTCTGTTTAACCCAGACATTGCCGGACTGCAGTGT
CAGCACTACATCCAGGCTCTGCAGAGCGAGGCGCACCAGGCTCTTAATGA
ACATGTCAAACTGATTCACCGAGGAGACACCACGAGGTTTGCCAAACTCT
TCATTGCCCTCTCAATGCTACGATCCATCAACGCCAATGTCGTGGCAGGGCT
CTTCTTCAGACCTGTCATTGGCACAGTAAACATGGAAGAACTTCTCCTCGA
GATGTTTTACGGAAAGTAG

Protein (evm.model.LG22.331) Length：290

MHGSEQKKRHLFQSGRSASMAHSESCECGGERRHPSILYSLLKKESAPGASAR
APRSARFAVAARTCACGVARPVALRSPHATCAAASAVLAKTLRFVKSVPCFGE
LPACDQRALVRSGWAPLLVLGMAQDGLDFETRESAEPSMLQRLLTGAHERPA
SPHDHGAHVINGGGGVALAEAQGIKAFLSKCWDLDISTKEYAYLKGAILFNPD
IAGLQCQHYIQALQSEAHQALNEHVKLIHRGDTTRFAKLFIALSMLRSINANV
VAGLFFRPVIGTVNMEELLLEMFYGK

nanos1

Gene ID：evm.TU.LG18.427strand：-start：13963549end：13978279length：14731

Transcript ID：evm.model.LG18.427 start：13963549 end：13978279 length：936

CDS(evm.model.LG18.427)Length:936 type:complete

ATGGACCCAGCAGACAAGGGGGAAAGGCTTATAACCAGCAGCCTCGCGCG
CAGCCCGAAATACCCAAGACAAGCGCTGGACCTGACTACATCTGTTAGCTA
CTTGCCGTTTCAATTGGGACGGAATATTTATTATCCGTTTTATCCTGCACAGA
GAGGCGCCGTCGCGCCGCTCGGGAGTCAGGGGAAAAGCGAGATGGATTTT
TTAAACCACAGCTACTTAAGCGCGCGCACGTCCTACGACTACACGTTCAAC
TTCTGGAACGACTATCTGGGCCTGTCCACGCTGGTGACGCAGAGCAGCAA
GCGCGGTCCCGGCGGCGGAGCGAGCCCCAACTCCATCACCGAATCTCTGA
AAGCCACACTGGGCCTGGATGACCCTGTGCCGTGCCCATGCGCCGCGCAC
TTGGACTACTGCTGCTGCTCATCCTCGTGCTGCTCCTCTTCCTCGTCGTGCG
GCTGCTGCTGCCCTCCCGCAAGCCCGCCGCCGCCTTCCCTAGTTGAGCTCA
AGGAGCGCTTCTCGGCGCTCGGGCCGTTCCGTAGCCACGGCGCAGGGATT
GTAGGGCATGAGCGCGACGCGGGCTTCGGAGGAGGAGGAGGAGGAGGAG
GAGGTGGAAGTTTCGCGGCTTTCGAGCTGTTCGGCGCGGATAGGAAGGTG
CGCAAGACAGCTGCGGCGCGCGCCAAGCAGGAGCCCAAGATCTGCGTGTT
CTGTCGGAATAACGGCGCGCCTGAAGAGGTGTACGGCTCGCACGTGCTGA
AGGCGCCCGACGGCCGCGTGGTGTGCCCGATCCTGCGCGCATACACGTGC
CCGCTGTGCAGCGCCAACGGCGACAACGCACATACCATCAAGTACTGTCC
ACTCTCCAAGGAGCAGCCCGCGCCTCGAGCGCTCAAAGGAGGCCGGGCTG
TGGGCGGTAAGCGCGTCAAAATCTTCTAA

Protein (evm.model.LG18.427) Length：311

MDPADKGERLITSSLARSPKYPRQALDLTTSVSYLPFQLGRNIYYPFYPAQRGA
VAPLGSQGKSEMDFLNHSYLSARTSYDYTFNFWNDYLGLSTLVTQSSKRGPG
GGASPNSITESLKATLGLDDPVPCPCAAHLDYCCCSSSCCSSSSSCGCCCPPASP
PPPSLVELKERFSALGPFRSHGAGIVGHERDAGFGGGGGGGGGGSFAAFELFG
ADRKVRKTAAARAKQEPKICVFCRNNGAPEEVYGSHVLKAPDGRVVCPILRA
YTCPLCSANGDNAHTIKYCPLSKEQPAPRALKGGRAVGGKRVKIF

ar

Gene ID：evm.TU.LG08.21strand：.start：4453979end：4539864length：85886

Transcript ID：evm.model.LG08.21 start：4453979 end：4539864 length：2571

CDS (evm.model. LG08.21) Length:2571 type:complete

ATGGAAGCTCGGTTCGGTTTAGGCGGAGTTTTGATTCTCCGTTCCGAACG

GTGCGTCCGCATGGCGCGCGAGACATCGGAGCGGCGGGAGAAGTTGTTCC

GCTGGCGGAGATGTGGGACGGAGAGTGGAGAGACGCGCGCGGGATTAGC

AGCACCGGCGGCGGCGGAGGAGGAGGAAGTGGGGGAGGAGGAGGAGGA

GGAGGAGGGACAGCGCGCTCCGTTACAGCGGCGCTCGCGCGGGACGGAA

GAGAAACCTCGAGTTGCACGGAGGCCCGCGCCGCCACCACCACGCTGAG

GGAGCTGCTCTGTCCGGACACCGGATACAACAACAACAACCACAGCAGCA

GAAGCAGCAGCGACAACCACAACACCGCTCCGAGAGCACGCGGCAGCGC

GCGCGAGCTGACAGCCGGCGGTGGTGGTGACACGAGCGCCGACCCGAGC

TGCTGCTGTTCCGGAAGCACCGGCACGACCACCAGCACCACGACCATCTC

TGAGACGGCGCGCGAGCTGTGCAAAGCCGTGTCCGTGTCGCTGGGGCTGG

CCATGGACCCGCACGCGCACCAGGACGGGAACAGGAGCTCGGACTTTTTC

GAAGTGCGCCGGAGGAAGGAGGAGGAAGAGGAGCGGGTGCACGCGCCA

AGCGACGACAGGAACAGAAGGATCAGCAGCAGCAGCGCGAGCGCAGGTG

GAGGTTGCGCCTCAGGGGAACGTGAGGGACGCGCGCCGCACGTGCACGG

CGACAACAACAAACTGCTCGAGATGTTCAAAAGTGGGGGCGAGGAGAAC

CTGATGATGATGACGACGATACAGATGGACAACGTTCGCGCGCATCACCAT

ACGTCAGTGATAACGGAAAATTCGGAGTTCGGACCGGGCGTCGCGAGCCT

GATGAGTTCCAAAAGCGCGAGCGCGGCGGCGGGTTCGTCGTCTTCCTCTTC

GTCCTGTCAGTTCCAACAACAACAACAACTACACGACCCGCAGGTAAACT

TCGAGCACGCGCCGCCCGTGCACGACAGGTACGGTGATGATTACTACTACT

ACTGGCCGCGCTACAGCAACGTGCGCGTGAAGTCCGAGGCCGTGCCGCCA

TCGGTGACTCGCTACTGTCACGACGCTGCGCAGTACGGGAATCCCACGTGC

ACCGCCTCCTCCGCCGGACTGGACGCGCCGCTCATCTGCAGCCCGTACGAG

TACGACTACGGCGCGCGGACCGGACCGGGACCGGCTCTCAGCGCCGCGCA

CGAGTCATGGTACCACCAGAGCGGCGCCGTGTTACCCCGCGTGTCCTACCC

AACCGCCACGTGCATGAAGAACGAAGTCGGGGAATGGCTGGATGTGAGCA
CGATGCAGGACGGCAGGTTGGAGAGTGCGAGGGAGGTGTTCCCCATGGAG
TTTTTCTTCCCTCCGCAGCGCACCTGCCTGATCTGCTCTGATGAAGCTTCCG
GTTGCCATTACGGAGCTCTGACCTGCGGCAGCTGCAAAGTGTTTTTCAAAA
GAGCGGCTGAAGGGAAGCAGAAGTATCTGTGTGCGAGCAGGAACGACTG
CACCATCGATAAGCTGAGGAGGAAGAACTGTCCGTCATGCCGTCTGAAGA
AGTGCTTTGAGGCAGGAATGACTCTCGGAGCTCGTAAACTGAAGAAGATT
GGTCACCTGAAGGTTCCTGAAGAGGAGGGCCCGGTTTCATCTATGCCGTCC
GGTTCCGGTTCGGTCGAGATGATCCGGAATCCATCACCCAAAGCGAGTCTG
ACGCTCCACTCACAGCTCGTCTTCCTCAGCATCCTGGAGTCCATCGAGCCT
GAAGTGGTGAACGCCGGTCATGACCACGCACAGCCCGACTCCGCTGCTGC
CCTCCTGACCAGCCTTAACGAGCTTGGAGAGAGGCAACTCGTTAAGGTGG
TGAAATGGGCCAAAGGACTGCCAGGATTCCGTAACCTCCACGTGGACGAT
CAGATGACGATGATCCAACATGCCTGGATGGCTGTGATGGTCTTCGCTCTC
GGATGGAGATCGTACAAAAACGTCAACGCTCGCATGCTCTACTTCGCTCCT
GACCTGGTCTTCAATGATCGGAGGATGCACATCTCCAGTATGTATGAACACT
GTCTGCAGATGAAACACTTGTCGCAGGAGTTCGTTTTGCTGCAGGTCACAC
AGGAGGAGTTTCTCTGCATGAAGGCCCTCCTCCTCTTCAGCATCATTCCTGT
GGAGGGATTGAAGAGTCAGAAGTACTTTGATGAGTTGAGGTTGTCGTATAT
AAACGAACTCAATCGTCTGATTAATTACGGCAGGAAGAGTAACTGTGCACA
GCGTTTCTACCAACTGACCAGACTGATGGACTCACTGCAGCCGATTGTCCG
GAAGCTTCACCAGTTCACCTTTGACCTTTTCGTTCAGGCTCAGTCTCTGCC
CACGAAGGTCAGCTTCCCGGAGATGATCGCCGAAATCATCTCAGTGCAGGT
ACCAAAGATCCTCGCCGGCCTGGCCAAGCCCATCCTGTTCCACAAGACTGT
GACTCCGCCCTCTCCCGCACCCATTCTGGTCACACCCACTCAGCCAGACCT
GGCCTGA

Protein (evm.model.LG08.21) Length：856

MEARFGLGGVFDSPFRTVRPHGARDIGAAGEVVPLAEMWDGEWRDARGISS
TGGGGGGGSGGGGGGGGGTARSVTAALARDGRETSSCTEARAATTTLRELLC
PDTGYNNNNHSSRSSSDNHNTAPRARGSARELTAGGGGDTSADPSCCCSGST

GTTTSTTTISETARELCKAVSVSLGLAMDPHAHQDGNRSSDFFEVRRRKEEEE

ERVHAPSDDRNRRISSSSASAGGGCASGEREGRAPHVHGDNNKLLEMFKSGG

EENLMMMTTIQMDNVRAHHHTSVITENSEFGPGVASLMSSKSASAAAGSSSS

SSSCQFQQQQQLHDPQVNFEHAPPVHDRYGDDYYYYWPRYSNVRVKSEAVP

PSVTRYCHDAAQYGNPTCTASSAGLDAPLICSPYEYDYGARTGPGPALSAAHE

SWYHQSGAVLPRVSYPTATCMKNEVGEWLDVSTMQDGRLESAREVFPMEFF

FPPQRTCLICSDEASGCHYGALTCGSCKVFFKRAAEGKQKYLCASRNDCTIDK

LRRKNCPSCRLKKCFEAGMTLGARKLKKIGHLKVPEEEGPVSSMPSGSGSVE

MIRNPSPKASLTLHSQLVFLSILESIEPEVVNAGHDHAQPDSAAALLTSLNELG

ERQLVKVVKWAKGLPGFRNLHVDDQMTMIQHAWMAVMVFALGWRSYKNV

NARMLYFAPDLVFNDRRMHISSMYEHCLQMKHLSQEFVLLQVTQEEFLCMK

ALLLFSIIPVEGLKSQKYFDELRLSYINELNRLINYGRKSNCAQRFYQLTRLMD

SLQPIVRKLHQFTFDLFVQAQSLPTKVSFPEMIAEIISVQVPKILAGLAKPILFH

KTVTPPSPAPILVTPTQPDLA
