# Supplementary material for: Gonadal Transcriptome Analysis and Sequence Characterization of Sex-Related Genes in Cranoglanis bouderius
Source: Int J Mol Sci. 2022 Dec 13;23(24):15840. doi: 10.3390/ijms232415840 (PMC9779447; doi:10.3390/ijms232415840)
Supplement: Supplementary file 1 [file ijms-23-15840-s001.zip › table S3 Sequences of primers of sex-related gene.pdf]

Supplemental Table S3. Sequences of primers.

Primers	Sequences (5’ →3’)
β-actin-F	ATGAAGATCCTGACCGAGAGAGGCTAC
β-actin-R	GCCAATGGTGATGACCTGTCCG
amh-F	CTTGGGCAGTAGAAAGAGCA
amh -R	GCATGGTTGTTACCGTTGAT
dax1-F	GAGACACCACGAGGTTTGCC
dax1-R	GAAGAGCCCTGCCACGACA
nanos1-F	CCCCAACTCCATCACCGAATC
nanos1-R	CGCACGACGAGGAAGAGGA
ar-F	GGCTGGATGTGAGCACGAT
ar-R	CGCTGCGGAGGGAAGAAA

F: (sense primer); R: (antisense primer).
