# Supplementary figures and images for: Gonadal Transcriptome Analysis and Sequence Characterization of Sex-Related Genes in Cranoglanis bouderius
Source: Int J Mol Sci. 2022 Dec 13;23(24):15840. doi: 10.3390/ijms232415840 (PMC9779447; doi:10.3390/ijms232415840)

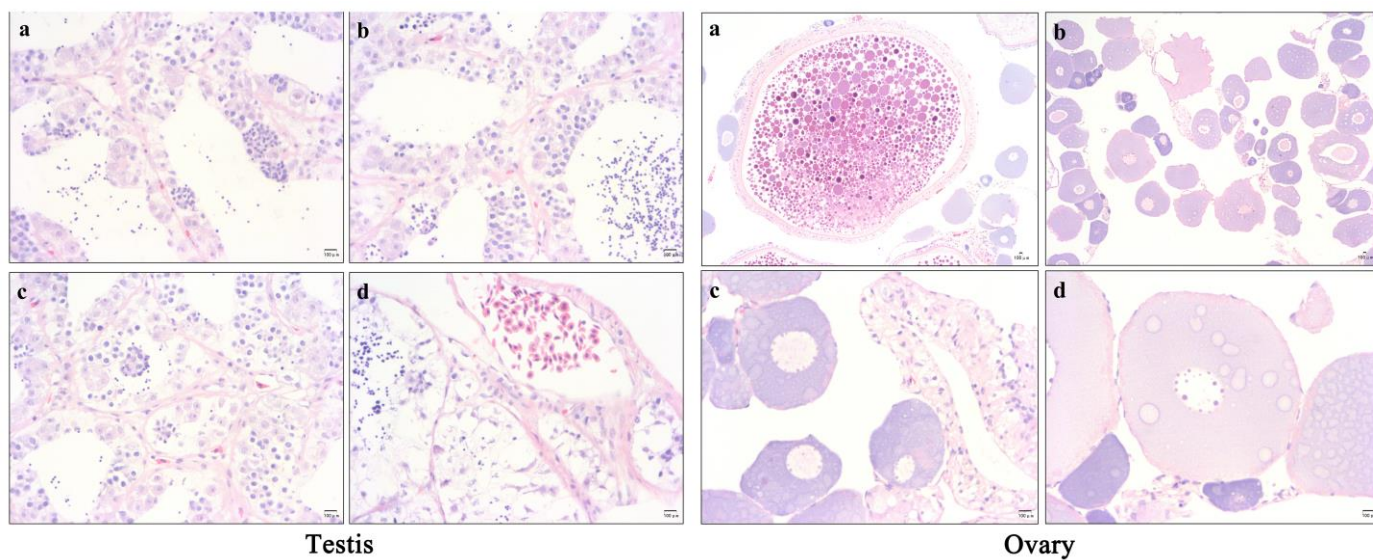

**Figure S1.** Histological structure of the testis and ovary of *Cranoglanis boudierius*

Supplement: Supplementary file 1 [file ijms-23-15840-s001.zip › figure S1 Histological structure of the testis and ovary of Cranoglanis bouderiusr.pdf]
